# Indirect treatment comparisons including network meta-analysis: Lenvatinib plus everolimus for the second-line treatment of advanced/metastatic renal cell carcinoma

**DOI:** 10.1371/journal.pone.0212899

**Published:** 2019-03-05

**Authors:** Gabriel Tremblay, Heather J. McElroy, Tracy Westley, Genevieve Meier, Derek Misurski, Matthew Guo

**Affiliations:** 1 Department of Health Economics, Purple Squirrel Economics, New York, New York, United States of America; 2 Department of Health Economics, Covance Pty Ltd, Macquarie Park, NSW, Australia; 3 Department of Oncology, Eisai Incorporated, Woodcliff Lake, New Jersey, United States of America; The University of Warwick, UNITED KINGDOM

## Abstract

**Background:**

In the absence of clinical trials providing direct efficacy results, this study compares different methods of indirect treatment comparison (ITC), and their respective impacts on efficacy estimates for lenvatinib (LEN) plus everolimus (EVE) combination therapy compared to other second-line treatments for advanced/metastatic renal cell carcinoma (a/mRCC).

**Methods:**

Using EVE alone as the common comparator, the Bucher method for ITC compared LEN + EVE with cabozantinib (CAB), nivolumab (NIV), placebo (PBO) and axitinib (AXI). Hazard ratios (HR) for overall survival (OS) and progression-free survival (PFS) estimated the impact of applying three versions of the LEN+EVE trial data in separate ITCs. Last, to overcome exchangeability bias and potential violations to the proportional hazards assumption, a network meta-analysis using fractional polynomials was performed.

**Results:**

Bucher ITCs demonstrated LEN + EVE superiority over EVE for PFS, indirect superiority to NIV, AXI, and PBO, and no difference to CAB. For OS, LEN + EVE was superior to EVE and indirectly superior to PBO, applying original HOPE 205 data. Using European Medicines Agency data, LEN + EVE was directly superior to EVE for OS. Fractional polynomial HRs for PFS and OS substantially overlapped with Bucher estimates, demonstrating LEN+EVE superiority over EVE, alone, NIV, and CAB. However, there were no statistically significant results as the credible intervals for HR crossed 1.0.

**Conclusions:**

Comparing three Bucher ITCs, LEN + EVE demonstrated superior PFS when indirectly compared to NIV, AXI, and PBO, and mixed results for OS. While fractional polynomial modelling for PFS and OS failed to find statistically significant differences in LEN + EVE efficacy, the overall HR trends were comparable.

## Introduction

In the United States, approximately 63,990 new cases will occur and 14,400 people will die from kidney and renal pelvis cancer in 2017 [1]. Renal cell carcinoma (RCC) is the most prevalent form of kidney cancer, diagnosed in approximately 90% of cases [2]. Treatment for advanced/metastatic RCC (a/mRCC) typically consists of single agents, however the combination of lenvatinib plus everolimus (LEN+EVE) demonstrated significant improvements in progression-free survival (PFS) compared to EVE as monotherapy among second-line a/mRCC patients (HOPE 205 trial, NCT01136733, Motzer et al. 2015) [3]. Currently, there are no other direct, head-to-head clinical trials comparing combination LEN + EVE to active comparators. Therefore, indirect treatment comparison (ITC) may be useful for informing of LEN+EVE efficacy in the absence of clinical trials.

The aim of this study was to compare overall survival (OS) and PFS efficacy outcomes among relevant second-line a/mRCC drug therapies: LEN+EVE to axitinib (AXI), cabozantinib (CAB), EVE, nivolumab (NIV), placebo (PBO) and by applying different methods of ITC analysis. Our objectives were: (1) Compare Bucher ITC’s for three different versions of HOPE 205 data: original trial data (Motzer 2015) [3], extended OS data (European Medicines Agency 2016) [4], and extended OS data with re-stratification of the models (FDA 2016) [5] (2) Compare these efficacy estimates to network meta-analysis (NMA) results using fractional polynomials, where the proportionality of hazards can vary.

## Materials and methods

A systematic literature review was conducted to gather relevant data on second-line a/mRCC drug therapies as described in the Supporting Information.

### Data sources for LEN+EVE efficacy: Three versions of HOPE 205 trial data

The original study from Motzer et al. (2015) reported hazard ratios (HR) for OS and PFS of LEN+EVE versus LEN and LEN+EVE versus EVE among participants with prior VEGF therapy. Additionally, more mature OS data were submitted to the European Medicines Agency (EMA) and the Food and Drug Administration (FDA), along with PFS outcomes reassessed by independent reviewers [3–5]. Furthermore, the FDA accepted the OS and PFS hazard models with different values for the stratification factors compared to the EMA submission and original trial analysis. Consequently, three sets of efficacy results comparing LEN+EVE to EVE are summarized in Table 1.

**Table 1:** LEN+EVE data sources for three separate ITCs applying hazard ratios.

Submission	LEN+EVE vs:	PFS end date	OS end date	HR OS	HR PFS
**1. Motzer 2015**	EVE	June 2014	Dec. 2014	0.51 (0.30;0.88)*	0.40 (0.24;0.68)*
**2. EMA 2016**	EVE	June 2014	July 2015	0.59 (0.36;0.97)*	0.50 (0.26;0.79)*
**3. FDA 2016**	EVE	June 2014	July 2015	0.67 (0.42;1.08)	0.37 (0.22;0.62)*

*Indicates reported significance with a 95% Confidence Interval; LEN, lenvatinib; EVE, everolimus; PFS, progression-free survival; OS, overall survival; HR, Hazard Ratio; EMA, European Medicines Agency; FDA, Food and Drug Administration.

#### 1. Original Motzer publication reporting post-hoc OS trial data (December 2014)

Trial investigators reviewed MRI and CT scans for disease progression. PFS data ended in June 2014, while OS data were extended to December 2014. HRs with 95% confidence intervals (CIs) were estimated for PFS and OS using stratified Cox regression models and the Efron method for tied events. Patients were stratified by hemoglobin level and corrected serum calcium. Additional trial characteristics are listed in S5–S7 Tables, in the Supporting Information.

#### 2. EMA [4] request for independent radiological review (IRR) and OS (July 2015)

For the PFS data, the EMA requested independent reviewers blinded to treatment. For OS, data were extended to July 2015. Statistical methods remained the same and the independent reviewer-led results for PFS were published in a follow-up Motzer et al article (2016) [6]. The updated OS results were included in EMA online product information [4].

#### 3. FDA re-analysis of EMA data for PFS (June 2014) and OS (July 2015)

While the FDA accepted the same data as the EMA request, the FDA required a change in stratification factors based on the data recorded in the randomization system instead using case report forms. After this amendment, the proportionality of hazards assumption was still considered satisfied.

### Proportionality of hazards assumption

Due to the three different sets of OS efficacy results for the various submissions, it was decided to separately apply versions 1, 2, and 3 (Table 1) of the HOPE 205 trial data into three separate ITCs for comparison. To obtain comparative efficacy results without risk of bias from proportionality violations, a NMA applying parametric fractional polynomials was conducted. After reviewing the a/mRCC studies being compared, this NMA fractional polynomial technique was requested by the Evidence Review Group (ERG) of the National Institute of Healthcare and Excellence (NICE). Based on previous findings from NICE assessment committees for CAB (GID-TA10075 [7]) and NIV (TA417 [8]), the ERG advised to consider AXI as having similar efficacy to EVE. This network omitted the RECORD-1, TARGET, and AXIS trials, whose participants did not have prior anti-VEGF therapy, were of lower risk, may have crossed over to investigational treatments within RECORD-1 and TARGET [9–11]. Thus, a more homogeneous network of trials with greater across trial comparability would be analysed.

### Statistical analysis for the Bucher indirect treatment comparisons

The ITCs were performed in Microsoft Excel 2010. Data and results were verified by two quality control reviewers. Efficacy outcomes from the SLR that entered into the ITCs included published HRs for PFS and OS, with two-sided 95% CIs on the natural log scale. Standard errors (SE) were calculated by subtracting HR from the 95% confidence limit. For trials reporting patient crossover (TARGET and RECORD-1), adjusted results available from publications entered the ITCs. ITCs used the Bucher method with EVE as the common comparator and proportionality of hazards within trials was assumed [12].

### Statistical analysis for the network meta-analyses with fractional polynomials

First, survival data were digitally extracted from the published Kaplan-Meier curves for CHECKMATE-025 and METEOR using UnGraph software package [13–15]. Where insufficient details were available on the published Kaplan-Meir curves (e.g., overlapping censor symbols, number censored not reported [CHECKMATE-025]), the method of Guyot et al (2012) was used [16]. The estimated survival functions apply a wide family of models including Weibull and Gompertz distributions in a method described by Jansen (2011) [17]. First order and second order polynomials using fixed effects estimated the treatment effect with multiple parameters using the Markov Chain Monte Carlo (MCMC) method in WinBugs [18]. Two chains were run for 50,000 iterations and discarded as “burn-in,” and then the model was run for a further 50,000 iterations for inference. Non-informative priors were used, and convergence was confirmed with diagnostic plots and the Gelman-Rubin statistic. The powers for the fractional polynomials were chosen from the set: -2, -1, -0.5, 0, 0.5, 1, and 2. The Deviance Information Criterion (DIC) was used to compare the goodness of fit.

## Results

### Systematic literature review

**Fig 1:**
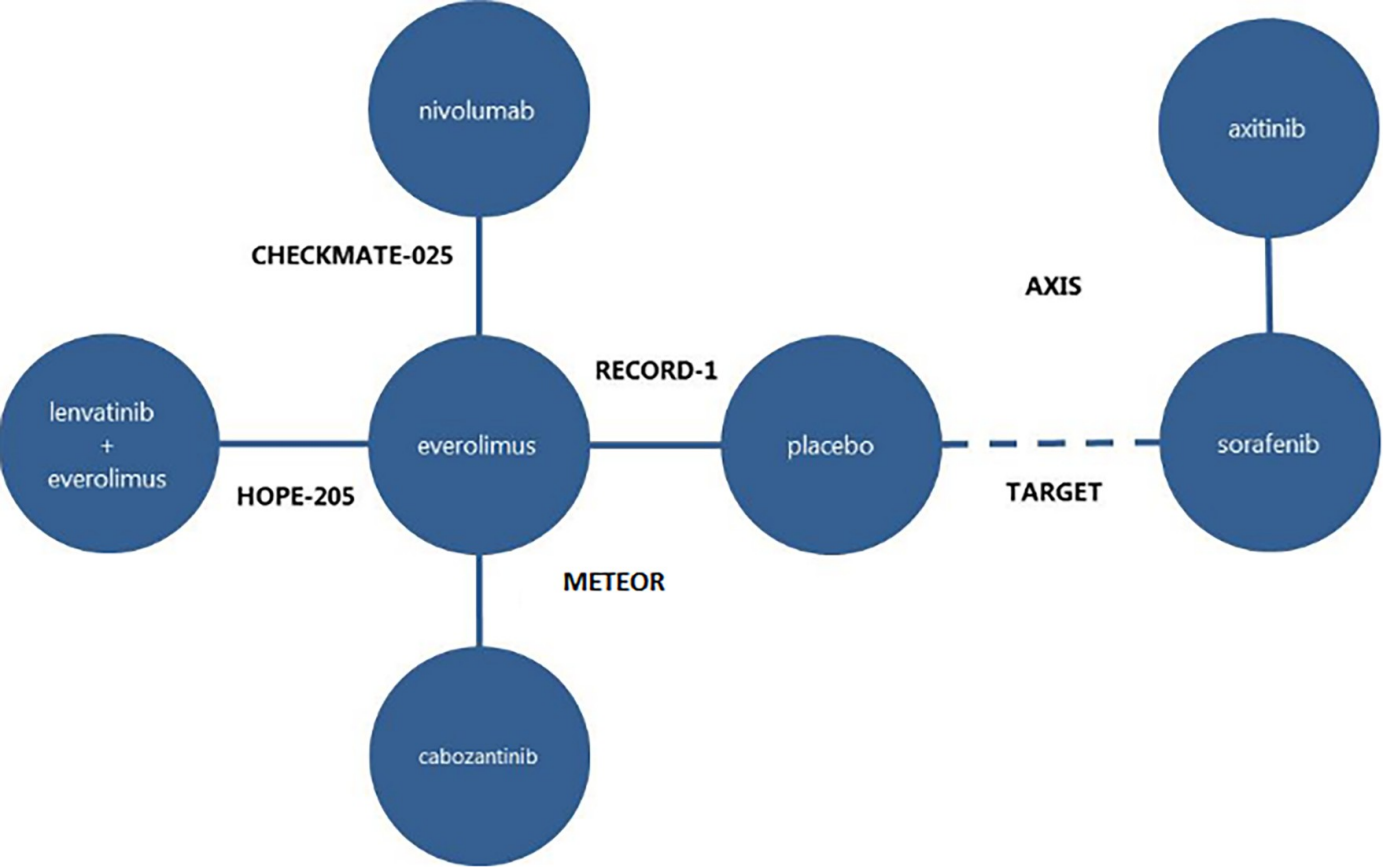
Network of trials included in the Bucher ITCs for a/mRCC.

Final selection of included and excluded studies is described in the Supporting Information. To enable indirect comparison of AXI with the HOPE 205 trial, a multi-step ITC was designed, connected by adding the TARGET trial (SOR vs PBO; Escudier 2009) [9]. This resulted in six studies for ITC, as listed in Table 2 and illustrated in Fig 1.

**Table 2:** Baseline characteristics comparison of patients in the clinical trials.

	Reference	Median Age (years)	Line of Therapy*	Gender (% Male)	ECOG (0,1)	MSKCC risk Favourable/ Intermediate/ Poor
**HOPE-205 NCT01136733 (Motzer et al 2015) [3]**	LEN + EVE (N = 51) vs EVE (N = 50)	61 vs 59	Second line	69% vs 76%	53,47 vs 56,44	**LEN+EVE:** 24%/37%/39%; **EVE:** 24%/38%/38%
**AXIS NCT00678392** **(Motzer et al 2013) [11]**	AXI (N = 361) vs SOR (N = 362)	61 vs 61	Second line	73% vs 71%	54,45 vs 55,44	**AXI:** 28%/37%/33%; **SOR**: 28%/36%/33%
**METEOR NCT01865747** **(Choueiri et al 2015) [14]**	CAB (N = 330) vs EVE (N = 328)	63 vs 62	Second line	77% vs 73%	68,32 vs 66,34	**CAB:** 45%/42%/12%; **EVE:** 46%/41%/13%
**CHECKMATE-025 NCT01668784** **(Motzer et al 2015) [15]**	NIV (N = 410) vs EVE (N = 411)	62 vs 62	Second line	77% vs 74%	**KPS:** *NIV/EVE* 0/90-100 68%/65% 1/70-80 32%/35%	**NIV:** 35%/49%/16%; **EVE:** 36%/49%/15%
**RECORD-1 NCT00410124** **(Motzer et al 2008) [10]**	EVE (N = 139) vs PBO (N = 277)	61 vs 60	Second line	78% vs 76%	**KPS:** *PBO/EVE* 0/90-100 68%/63% 1/70-80 33%/36%	PBO: 28%/57%/15%; EVE: 29%/56%/14%
**TARGET NCT00073307** **(Escudier et al 2009) [9]**	SOR (N = 452) vs PBO (N = 451)	58 vs 59	First line	70% vs 75%	49,49 vs 46,52	**PBO:** 51%/49%/-; **SOR:** 52%/48%/-

ECOG, Eastern Cooperative Oncology Group performance status; MSKCC, Memorial Sloan-Kettering Cancer Center; KPS, Karnofsky performance status; LEN, Lenvatinib; EVE, Everolimus; AXI, axitinib; CAB, cabozantinib; NIV, nivolumab; vs, versus

### Patient population for Bucher indirect treatment comparisons

Across the selected trials, patient characteristics (median age, gender, history of prior nephrectomy) were considered similar enough for ITC. As all patients were considered equally likely to be given any treatment in the network, adherence to transitivity was considered sufficient.

However, some differences still prevailed. The average patient in the HOPE 205 [15] and AXIS (AXI vs SOR; Motzer 2013) [11] trials had greater disease severity as measured by Eastern Cooperative Oncology Group (ECOG) performance status and Memorial Sloan-Kettering Cancer Center (MSKCC) risk level, while no patients in the TARGET trial had poor MSKCC risk. Additionally, patients in HOPE 205 could only have failed only one prior anti-VEGF, while approximately 30% of patients in the other EVE involved trials (CHECKMATE-025 [15], METEOR [14], and RECORD-1 [10]) had received more than one prior anti-VEGF therapy. The SOR trials (TARGET, AXIS) did not require failure of prior anti-VEGF therapy. All patients in TARGET and approximately 30% of patients in AXIS had no prior anti-VEGF therapies.

### Three indirect treatment comparisons using the Bucher method progression-free survival

The HOPE 205, CHECKMATE-025 and METEOR trials used RECIST 1.1 criteria to assess response, and RECORD-1, TARGET and AXIS used RECIST 1.0. Indirect estimates of HRs for PFS of LEN + EVE versus other treatments are presented in Table 3 below. Consistency across trials was assessed by visually examining the comparable median PFS in patients treated with EVE (Supporting Information S8 Table). However, there was a lack of direct evidence comparing LEN+EVE to either CAB, AXI, or SOR, which limited the ability to statistically report consistency. Graphically, this limitation is evident from the networks not containing any closed loops. Median PFS was higher in HOPE 205 than in the other three EVE involved studies even though patients in HOPE 205 were of higher risk and worse performance status. Across trials, the median PFS as well as the overall response rate (Supporting Information S8 and S10 Tables, respectively) did not vary greatly by method of radiologic review (INV versus IRR). When available, IRR-derived results entered the ITCs.

**Table 3:** Indirect treatment comparisons of progression-free survival: Hazard ratio (95% CI) for LEN + EVE versus comparators.

LEN + EVE vs	EMA (2016)	FDA (2016)	Motzer (2015)
**EVE**	0.45 (0.27;0.79)*	0.37 (0.22;0.62)*	0.40 (0.24;0.68)*
**Axitinib**	0.46 (0.23;0.91)*	0.38 (0.19;0.74)*	0.41 (0.21;0.80)*
**Cabozantinib**	0.78 (0.43;1.41)	0.64 (0.36;1.14)	0.69 (0.39;1.23)
**Nivolumab**	0.51 (0.29;0.89)*	0.42 (0.24;0.72)*	0.45 (0.26;0.78)*
**Placebo**	0.14 (0.08;0.26)*	0.11 (0.06;0.20)*	0.12 (0.07;0.22)*

*Indicates significance at a 5% significance level;

CI, confidence interval; LEN, lenvatinib; EVE, everolimus; EMA, European Medicines Agency; FDA, Food and Drug Administration.

For all versions of the HOPE 205 trial results, LEN + EVE was found to be superior to EVE alone, and indirectly superior to both NIV and PBO (Table 3). There were marginal differences in PFS between LEN + EVE and CAB. LEN + EVE was shown to be superior to AXI, though potential effect modification of none versus one prior VEGF-therapy may have biased results. However, the AXIS study did not report an estimate of the interaction term.

### Overall survival

Indirect estimates of OS HRs comparing LEN+EVE therapy versus other treatments, after adjustment for patient cross-over, are presented in Table 4. No statistically significant differences were observed for LEN+EVE versus NIV, LEN+EVE versus CAB, or LEN+EVE versus AXI. Compared to PBO, LEN+EVE was significantly superior applying the results from the Motzer publication (Dataset Version “1”) and ITT results for the placebo controlled trials RECORD-1 and TARGET (Supporting Information S11 Table). LEN+EVE was superior to EVE (based on the HOPE 205 trial and EMA’s mature OS dataset). Of note, more mature OS data did not result in improved LEN +EVE efficacy estimates. Furthermore, as with the analysis of PFS, the multi-step indirect comparison of LEN+EVE to AXI was in potential violation of the exchangeability assumption based on prior anti-VEGF status. OS results as reported by the individual trials are listed in Supporting Information S9 Table.

**Table 4:** Indirect treatment comparisons of overall survival: Hazard ratio (95% CI) for LEN + EVE versus other treatments.

LEN + EVE vs	EMA (2016)	FDA (2016)	Motzer (2015)
**EVE**	0.59 (0.36;0.97)*	0.67 (0.42;1.08)	0.51 (0.30;0.88)*
**Axitinib**	0.47 (0.15;1.50)	0.53 (0.17;1.68)	0.40 (0.12;1.30)
**Cabozantinib**	0.89 (0.52;1.53)	1.02 (0.60;1.72)	0.77 (0.43;1.38)
**Nivolumab**	0.81 (0.47;1.41)	0.92 (0.54;1.57)	0.70 (0.39;1.26)
**Placebo**	0.35 (0.11;1.07)	0.40 (0.13;1.22)	0.31 (0.10;0.97)*

*Indicates significance at a 5% significance level CI confidence interval;

LEN, lenvatinib; EVE, everolimus.

### Results from the network meta-analysis applying fractional polynomial survival curves

From the digital extraction of the published Kaplan-Meier curves, the proportional hazards assumption was found to be violated for PFS in CHECKMATE-025 and METEOR studies. The test for proportional hazards for PFS was not statistically significant for HOPE 205. However, the test was underpowered due to the sample size, and the diagnostic plots were similar to the other studies in violation. The log-cumulative curves suggest a change in hazards around seven weeks (~exp(2) = 7), which is likely to be due to interval censoring; the first protocol specified assessment of response was at eight weeks in all trials. The proportional hazard assumptions held for OS within the HOPE 205 and METEOR trials, but not for CHECKMATE-025.

**Fig 2:**
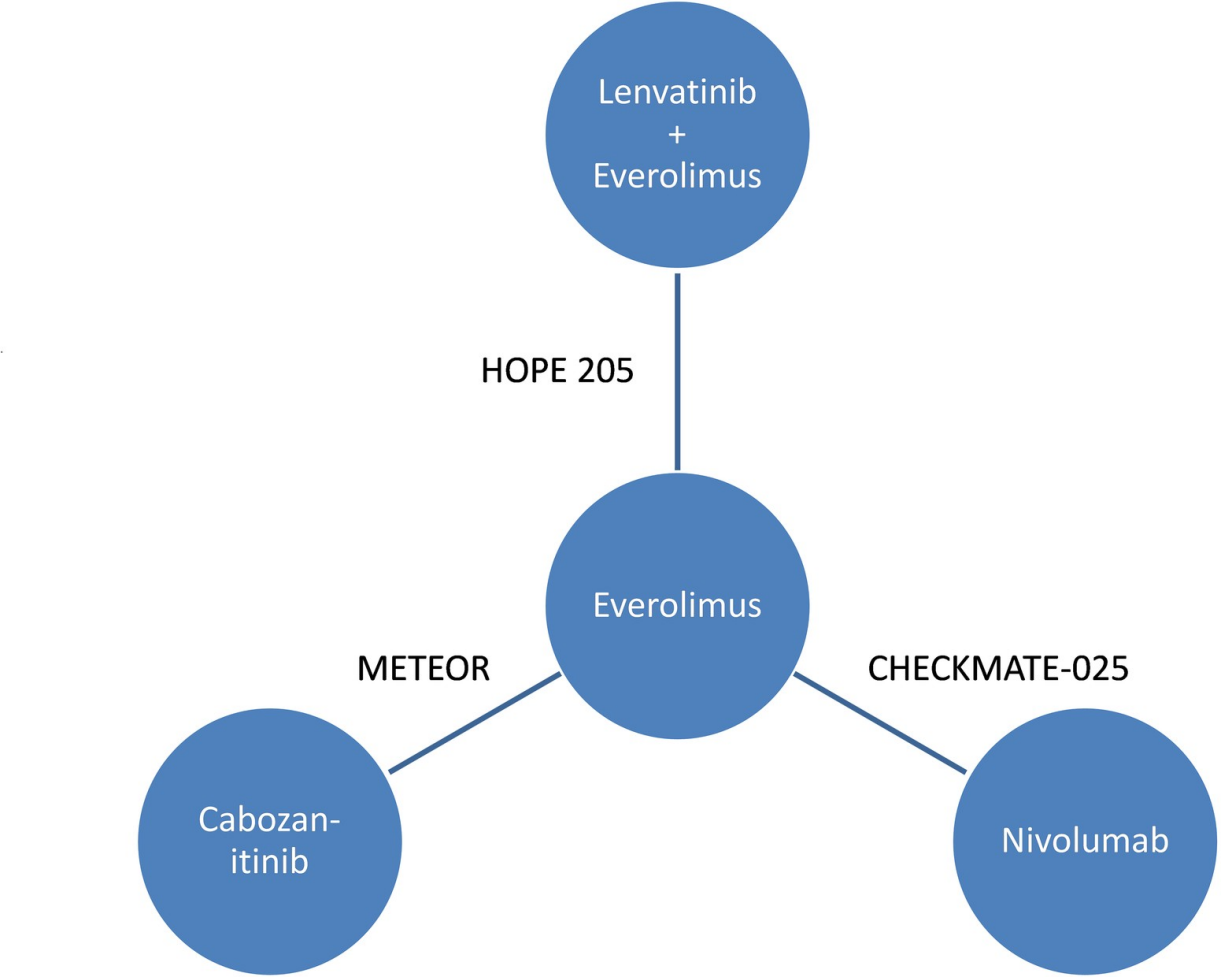
Network of trials for the NMA applying fractional polynomials.

With the assumption of similar efficacy between AXI and EVE monotherapy to increase network certainty, the final NMA included three trials for four treatment comparisons (Fig 2). INV assessment of PFS was available for CHECKMATE-025 (NIV vs EVE) [3], and IRR review was available for METEOR (CAB vs EVE) [14]. To avoid informative censoring of progressed patients from the HOPE 205 radiologic review, the investigator assessment of PFS was applied (Dataset Version “1”). For OS, the mature July 2015 data set (Dataset Version “2”) was applied. A second-order fractional polynomial (P1 = -2, P2 = -2) provided the best fit for PFS (DIC = 777.2). A first-order fractional polynomial (P1 = -1) provided the best fit for OS (DIC = 640.23), however visual inspection of the Kaplan-Meier overlay demonstrated an underestimated survival for NIV.

**Fig 3:**
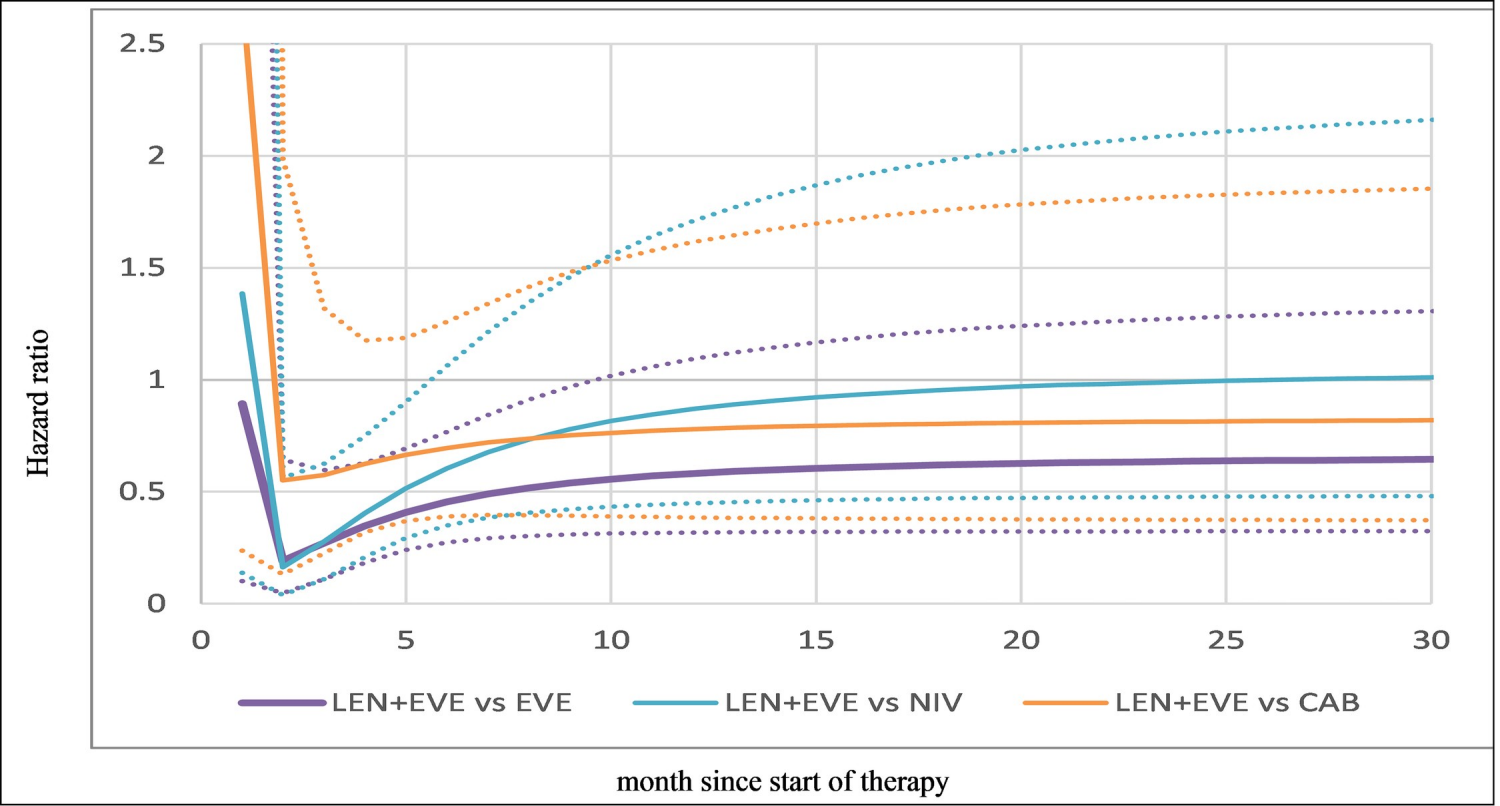
Hazard ratios over time for progression-free survival (fixed-effects second-order polynomial). CAB, cabozantinib; EVE, everolimus; LEV, lenvatinib; NIV, nivolumab; PFS, progression-free survival Notes: Solid line is median and dotted lines 95% credible intervals. Hazard ratios based on average estimates for everolimus over the three studies (μ_0_, μ_1_, μ_2_) per Jansen 2011 [17].

The hazard ratios over time (x-axis) for PFS resulting from this model are presented in Fig 3 and show that LEN +EVE is superior (HR < 1 on the vertical axis) to EVE monotherapy, CAB and NIV from about two months. However, the 95% credible intervals (dotted lines) cross 1 indicating these differences are not statistically significant. While the fixed-effect models for PFS fit the Kaplan-Meier data well, random-effects models were not explored due to expected instability due to a small network.

**Fig 4:**
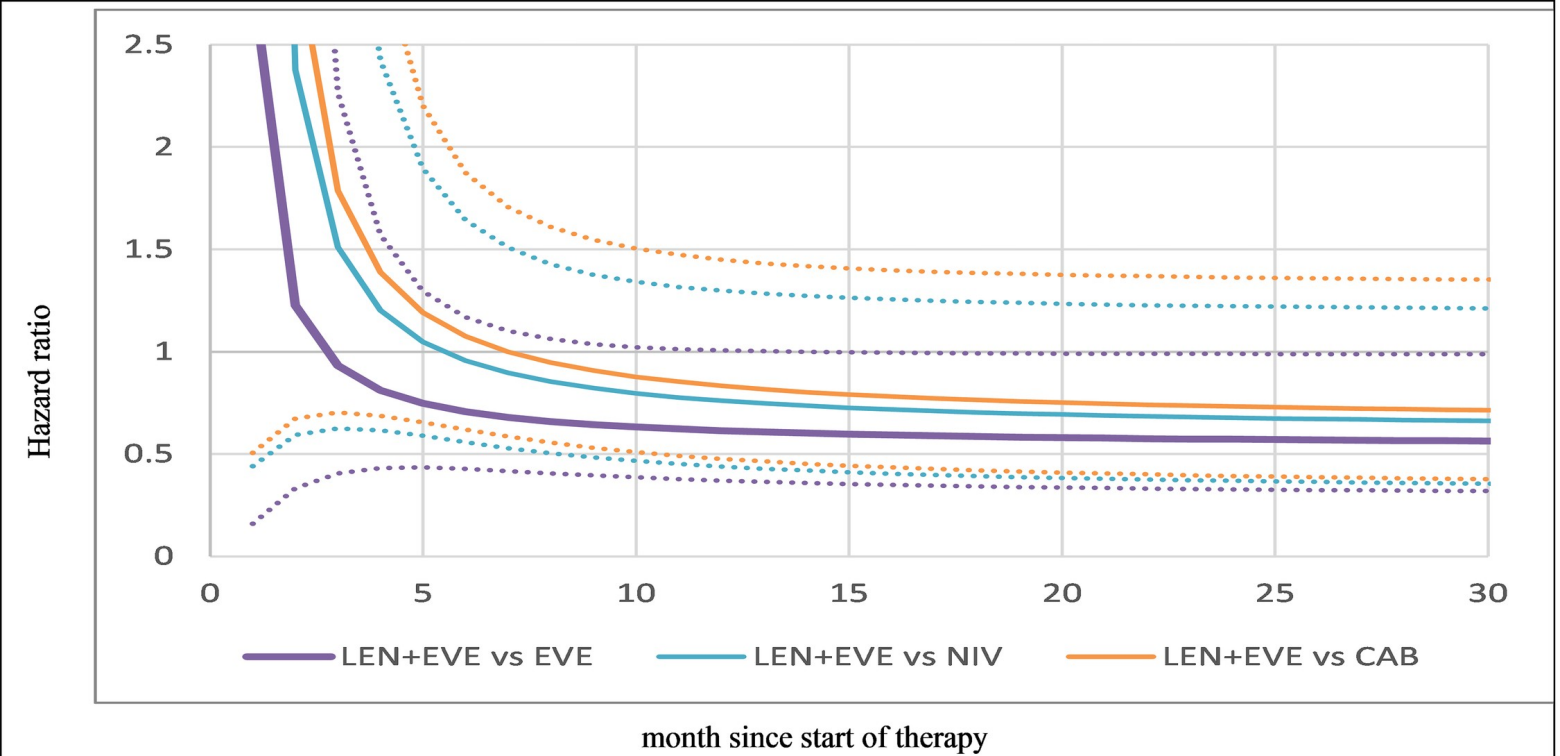
Hazard ratio over time for overall survival (fixed-effects first-order polynomial). CAB, cabozantinib; EVE, everolimus; LEV, lenvatinib; NIV, nivolumab; OS, overall survival; Notes: Solid line is median and dotted lines 95% credible intervals. Hazard ratios based on average estimates for everolimus over the three studies (μ_0_, μ_1_) per Jansen 2011 [17].

The hazard ratios over time (x-axis) for OS (Fig 4) show that LEN + EVE is numerically superior to EVE monotherapy from around two months, and CAB and NIV from approximately eight months. However, similar to PFS comparisons, the 95% credible intervals crossed 1 indicating these differences are not statistically significant. While first-order polynomials assume a monotonic association of treatment and effect, second-order polynomials were explored but did not result in better fitting models (as indicated by DIC). The HOPE 205 trial (32 events among 51 patients) was smaller than CHECKMATE-025 and METEOR (215 and 178 events for NIV and CAB, respectively). The second best fitting model (P1 = -2, P2 = 0) did not provide a good fit for LEN + EVE. While alternate second order models with higher powers (P1≥-1) provided better fit for LEN + EVE, there was not a better overall fit.

## Discussion

A summary of the three Bucher ITCs compared the efficacy of LEN + EVE to other second-line a/mRCC therapies when applying three different data sets derived from the HOPE 205 trial. Furthermore, results were compared to a Bayesian NMA relaxing the proportional hazards assumption. Based on the three Bucher ITCs, for PFS, the LEN + EVE combination was directly superior to EVE, and indirectly superior to NIV, AXI, and PBO. For OS, there were no statistically significant differences between LEN + EVE versus NIV, CAB, or AXI, and mixed results comparing EVE and comparing PBO. The fractional polynomial NMA resulted in comparable HR estimates, with LEN+EVE superiority over EVE, CAB, and NIV from two months for PFS and two (EVE) to eight (CAB) months for OS. However, with the added model parameters for time, the comparisons were not statistically significant.

### Impact of data sets on estimates

Three separate data sets emerged from the original July 2014 HOPE 205 (Motzer 2015) study as a result of requests from the FDA and EMA. The current analysis found ITC estimates to be marginally impacted by which version of the HOPE 205 trial data were applied. The post-hoc IRR estimates requested by the FDA resulted in higher hazard ratios compared to the EMA INV assessments (EMA INV results not presented) [5]. The IRR-derived estimates were, however, found to be complementary with the initial INV-derived estimates from the HOPE 205 original data. Likewise, a meta-analysis by Amit et. Al (2011) [19] and an FDA retrospective study in 2012 on INV versus IRR for PFS estimates reported a high degree of correlation between the two methods. Although there may be patient-level discrepancies between the two methods, regulatory authorities based their approvals of combination LEN +EVE by the overall population trends.

The EMA calculation of PFS and OS based on stratified data from case report forms is consistent with the HOPE 205 protocol (HOPE trial; Eisai SAP E7080-G000-205). Uniquely, the FDA stratified data recorded within the electronic randomization system. However, the comparability between electronic and paper administration for PROs was demonstrated in a 2015 meta-analysis by Muehlhausen et al [20]. While FDA-derived results had lower PFS point estimates and higher OS point estimates then original HOPE-205 results and EMA-derived results, the statistical trend was overall consistent across the three sets of results.

With the fractional polynomial method, HR trends reflected the Bucher-derived results. However, relaxing the proportional hazards assumption allowed HR to vary over time, and the increase in model parameters contributed to a decrease in power. Furthermore, with a small number of events in the HOPE 205 trial compared to the rest of trials in the reduced network, there may have been insufficient data to robustly fit one family of curves across the four treatments. Future analyses could extend the fractional polynomial modelling to permit a different family for each treatment.

### Limitations

The validity of any ITC is dependent on the exchangeability of patient baseline characteristics across the trials [21]. While HOPE 205 was an open label, Phase 2 study with a smaller sample size, the patient populations were comparable to CHECKMATE-025 and METEOR trials, with all patients having previously failed VEGF-therapy or SUN. Within the broader networks used for the Bucher ITCs, RECORD-1, AXIS, and TARGET studies were conducted in earlier time periods, with different patient populations, prior treatment failure history, and/or trial design features (crossover). For instance, the TARGET study found that additional prior therapy use was associated with worse OS and PFS. Therefore, the assumption of similar distributions of patient baseline characteristics (i.e., potential effect modifiers) required to produce robust ITC estimates may be violated, potentially limiting the relevance of the final results [22]. Furthermore, confounding effects from subsequent therapies (Supporting Information S7 Table) and continuing investigational treatment after progression may have increased within-trial uncertainty around the result estimates.

On the other hand, the fractional polynomial method omitted the TARGET and AXIS trial from the network, on the basis that AXI and EVE had comparable efficacy. Consequently, sources of bias from crossover and patient population differences were reduced. Furthermore, this smaller network consisted of all direct comparisons. Thus, Bucher-derived point estimates from this smaller set of trials would be comparable to the relevant results presented from the larger network. Consequently, comparison between Bucher and fractional polynomial results was appropriate.

## Conclusions

As evidence for the violation of proportionality assumption was not strong for OS, the Bucher ITCs and Bayesian fractional polynomial HR trends were similar when comparing LEN + EVE to other therapies in second-line a/mRCC. However, sources of potential biases and increased standard errors for both indirect methodological approaches ultimately contributed to wider confidence and credible intervals. For PFS, Bucher analysis found HRs demonstrating indirect superiority of LEN + EVE over AXI, NIV, and PBO, and for all versions of HOPE 205 trial data. For both methods, conclusions concerning LEN + EVE superiority for OS were challenged by the small trial size and number of events. Consequently, the fractional polynomial approach found numeric but not statistical significance in comparative effects. The nature of fractional polynomial modelling with added parameters for time-varying HRs produced substantially wider credible intervals, therefore assessment at each timepoint is also important to consider.

## Supporting information

S1 Text Methodological details on the SLR. (DOCX)

S1 Fig PRISMA flow diagram. (TIF)

S1 Table Inclusion/exclusion criteria for clinical evidence. (DOCX)

S2 Table Search terms for the clinical studies. (DOCX)

S3 Table List of trials included in the ITCs. *TARGET added after SLR, during the creation of the Bucher ITC network.
(DOCX)

S4 Table Excluded studies. (DOCX)

S5 Table Randomized controlled trials quality assessment. (DOCX)

S6 Table Baseline patient and disease characteristics. *The category *Unknown* signified unreported, missing, or unknown baseline characteristic data as reported by the original trial. ECOG, Eastern Cooperative Oncology group; MSKCC, Memorial Sloan-Kettering Cancer Center; NR, not reported; RT, radiotherapy; VEGF, vascular endothelial growth factor; HOPE 205, METEOR, TARGET and AXIS reported ECOG performance status; CHECKMATE-025 and RECORD-1 reported Karnofsky performance.
(DOCX)

S7 Table Subsequent therapies. NR, not reported; VEGF, vascular endothelial growth factor; LEN, lenvatinib; EVE, everolimus.
(DOCX)

S8 Table Progression-free survival as reported in the individual trials. CI, confidence interval; HR, hazard ratio; NR, not reported; PFS, progression free survival; VEGF, vascular endothelial growth factor; vs, versus. a Primary PFS population with 375 patients; b Protocol defined analysis of 769 patients; c At time of cross-over in 903 patients.
(DOCX)

S9 Table Overall survival as reported in the individual trials. CI, confidence interval; HR, hazard ratio; ITC, indirect treatment comparison; NR, not reported; OS, overall survival; RPSFT, rank preserving structural failure time; VEGF, vascular endothelial growth factor; vs, versus. a 98.5% confidence interval.
(DOCX)

S10 Table Overall response rate as reported in the individual trials. CI, confidence interval; n/N, number with event/number in efficacy population; NR, not reported; ORR, overall response rate; VEGF, vascular endothelial growth factor.
(DOCX)

S11 Table Overall survival applying ITT results from TARGET and RECORD-1. *Indicates significance at a 5% significance level CI confidence interval; LEN, lenvatinib; EVE, everolimus; EMA, European Medicines Agency; FDA, Food and Drug Administration.
(DOCX)

S1 File Previous presentation of partial results at the 2017 congress of the European society for molecular oncology. (PDF)

S2 File Previous presentation of partial results at the 2017 European congress of the international society for pharmacoeconomics and outcomes research. (PDF)

S3 File PRISMA checklist for NMA. (DOCX)
