# Supplementary material for: Indirect treatment comparisons including network meta-analysis: Lenvatinib plus everolimus for the second-line treatment of advanced/metastatic renal cell carcinoma
Source: PLoS One. 2019 Mar 5;14(3):e0212899. doi: 10.1371/journal.pone.0212899 (PMC6400440; doi:10.1371/journal.pone.0212899)
Supplement: S1 Text — (DOCX) [file pone.0212899.s001.docx]

## Systematic Literature Review

To search the clinical trials, included studies compared efficacy between at least one of the following drug treatments or placebo (PBO): LEN, EVE, axitinib (AXI), cabozantinib (CAB), nivolumab (NIV). The population, intervention, comparator, outcomes and study designs (PICOS) were defined to establish the inclusion/exclusion criteria described in Table S1.

Following PRISMA guidelines for systematic reviews, key biomedical databases were searched for English-language publications until October 2016: Embase (from 1947) and MEDLINE (from 1950), MEDLINE via PubMed, and the Cochrane Library (all entries) [1]. Additionally, trial registries (all entries) and the database EconLit (from 1988) were searched through November 2016. Within grey literature, websites including international health technology assessments (HTAs) as well as websites for international and U.S. cancer agencies and guidelines were searched. S2 Table lists the search terms for each database. Two independent reviewers screened titles and abstracts and met over disagreements. Full-text screening was performed by the same reviewers. The NICE methodology checklist for randomized controlled trials was used to assess the risk of biases (selection, performance, detection, or attrition) in selected studies [2]. The risk of bias was considered in the selection of the final studies.

Nine trials examining 10 treatment regimens for a/mRCC patients with at least one prior anti-VEGF therapy were considered from the SLR. However, four exclusions were made for trials not including the drugs of interest and if trials included patient populations too different from the rest of the studies.

**References**

1. Moher D, Liberati A, Tetzlaff J, Altman DG, Group P. Preferred reporting items for systematic reviews and meta-analyses: the PRISMA statement. PLoS Med. 2009;6(7):e1000097.

2. The guidelines manual. Appendix C: Methodology checklist: randomised controlled trials National Institute for Health and Care Excellence (NICE) 2012.
