# Supplementary figures and images for: Indirect treatment comparisons including network meta-analysis: Lenvatinib plus everolimus for the second-line treatment of advanced/metastatic renal cell carcinoma
Source: PLoS One. 2019 Mar 5;14(3):e0212899. doi: 10.1371/journal.pone.0212899 (PMC6400440; doi:10.1371/journal.pone.0212899)

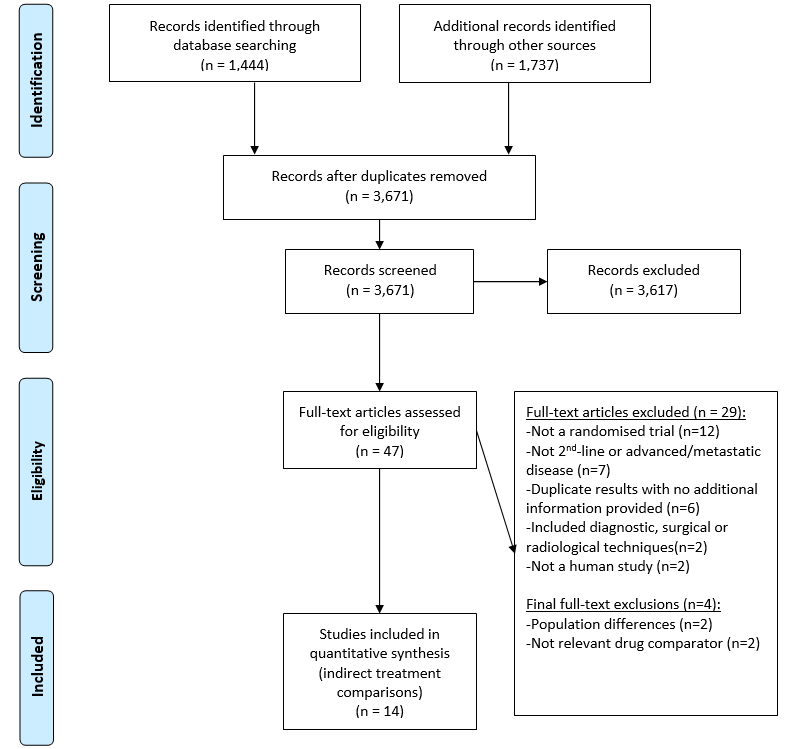

Supplement: S1 Fig — (TIF) [file pone.0212899.s002.tif]
