# Supplementary material for: Indirect treatment comparisons including network meta-analysis: Lenvatinib plus everolimus for the second-line treatment of advanced/metastatic renal cell carcinoma
Source: PLoS One. 2019 Mar 5;14(3):e0212899. doi: 10.1371/journal.pone.0212899 (PMC6400440; doi:10.1371/journal.pone.0212899)
Supplement: S1 Table — (DOCX) [file pone.0212899.s003.docx]

S1 Table: Inclusion/exclusion criteria for clinical evidence.

|  | Inclusion | Exclusion |
| --- | --- | --- |
| Population | - Patients >18 years old - Patients with mRCC who have received one previous systemic therapy | - Non-human - Children <18 years of age - Previously untreated patients |
| Intervention and Comparators | Any of the interventions below if administered as second line:   - Nivolumab - Cabozantinib - Everolimus - Temsirolimus - Axitinib - Pazopanib - Sorafenib - sunitinib - Bevacizumab - Aldesleukin | - Studies not including at least one of the comparators of interest listed in the inclusion criteria - Studies administering the comparators of interest as first-line therapy - Studies with surgery as a comparator |
| Outcomes | - Efficacy-related events - Safety-related events - Tolerability-related events | - Any not listed in the inclusion criteria |
| Study design | - Randomized Controlled Trials (Phase 2 and Phase 3) - Systematic Reviews (to be used for reference cross-checking only) | - Editorials - Notes - Comments - Letters - Reviews - Preclinical and Phase 1 trials - Retrospective studies including pooled post hoc analyses of clinical trials - Single arm or non-randomized studies - Case reports studies - Multi-Center - Meta-Analysis - Studies with less than 5 patients per arm |
