# Supplementary material for: Indirect treatment comparisons including network meta-analysis: Lenvatinib plus everolimus for the second-line treatment of advanced/metastatic renal cell carcinoma
Source: PLoS One. 2019 Mar 5;14(3):e0212899. doi: 10.1371/journal.pone.0212899 (PMC6400440; doi:10.1371/journal.pone.0212899)
Supplement: S2 Table — (DOCX) [file pone.0212899.s004.docx]

**S2 Table. Search terms for the clinical studies.**

| **Embase and Medline database search** | |
| --- | --- |
| DISEASE AREA – Advanced/metastatic renal cell carcinoma terms | |
| **1** | **("metastatic renal cell carcinoma" OR "metastatic renal cell cancer"):de** |
| **2** | **((metasta* OR "meta static" OR advanced) AND (rcc OR "renal cell" OR nephrocarcinoma OR "nephro carcinoma")):ti** |
| **3** | **((metasta* OR "meta static" OR advanced) NEAR/6 (rcc OR "renal cell" OR nephrocarcinoma OR "nephro carcinoma")):ab** |
| **4** | **(mrcc OR mccrcc):ti,ab** |
|  |  |
| DISEASE AREA – Renal Cell Carcinoma terms | |
| **1** | **kidney carcinoma/de** |
| **2** | **kidney cancer/de** |
| **3** | **("renal cell carcinoma" OR "renal cell cancer"):de** |
| **4** | **(("chromophobe cell kidney" OR hypernephroid OR "kidney cell" OR "kidney pelvic" OR renal OR "renal cell" OR "Collecting Duct" OR nephroid) NEXT/1 (carcinoma OR cancer)):ti,ab** |
| **5** | **("grawitz tumor" OR "grawitz tumour" OR hypernephroma OR "kidney adenocarcinoma" OR "kidney pyelocarcinoma" OR "renal adenocarcinoma"):ti,ab** |
| **6** | **kidney cell/de AND neoplasm/de** |
|  |  |
| DISEASE CHARACTERISTIC – Metastatic terms | |
| **1** | **metastasis/exp** |
| **2** | **advanced cancer/de** |
| **3** | **disease course/de** |
| **4** | **("cancer progression" OR "disease progression" OR "tumor progression" OR "tumour progression"):ti,ab** |
| **5** | **("metastatic disease" OR "metastatic cancer" OR "advanced disease" OR "advanced stage" OR "advanced stages"):ti,ab** |
| **6** | **(stage NEXT/3 (iiib OR iv OR 4 OR four)):ti,ab** |
| **7** | **((iiib OR iv OR ivth OR 4th OR fourth) NEXT/2 stage):ti,ab** |
|  |  |
| INTERVENTION – Vascular endothelial growth factor therapy terms | |
| **1** | **lenvatinib/de** |
| **2** | **(lenvatinib OR Lenvima OR "e 7080" OR e7080 OR "er 203492 00" OR "er203492 00"):ti,ab,tn** |
| **3** | **(417716-92-8 OR 857890-39-2):rn** |
| **4** | **cabozantinib/de** |
| **5** | **(cabozantinib OR Cabometyx OR cometriq OR "bms 907351" OR bms907351 OR "XL 184" OR XL184):ti,ab,tn** |
| **6** | **(1140909-48-3 OR 849217-68-1 OR 942407-59-2):rn** |
| **7** | **Nivolumab/de** |
| **8** | **(Nivolumab OR opdivo OR "bms 936558" OR bms936558 OR "mdx 1106" OR mdx1106 OR "ono 4538" OR ono4538):ti,ab** |
| **9** | **946414-94-4:rn** |
| **10** | **temsirolimus/de** |
| **11** | **(temsirolimus OR torisel OR "cci 779" OR cci779 OR "cell cycle inhibitor 779" OR "nsc 683864" OR nsc683864):ti,ab,tn** |
| **12** | **(162635-04-3 OR 343261-52-9):rn** |
| **13** | **everolimus/de** |
| **14** | **(everolimus OR affinitor OR afinitor OR certican OR "rad 001" OR "rad 001a" OR rad001 OR rad001a OR "sdz rad" OR votubia OR xience OR zortress):ti,ab,tn** |
| **15** | **159351-69-6:rn** |
| **16** | **pazopanib/de** |
| **17** | **(pazopanib OR armala OR votrient OR "GW 780604" OR GW780604 OR "gw 786034" OR "gw 786034b" OR "gw 786034x" OR gw786034* OR "sb 710468" OR "sb 710468a" OR sb710468*):ti,ab,tn** |
| **18** | **(444731-52-6 OR 635702-64-6):rn** |
| **19** | **sunitinib/de** |
| **20** | **(sunitinib OR sutent OR "pha 2909040ad" OR pha2909040ad OR "su 010398" OR "su 011248" OR "su 10398" OR "su 11248" OR su010398 OR su011248 OR su10398 OR su11248):ti,ab,tn** |
| **21** | **(341031-54-7 OR 557795-19-4):rn** |
| **22** | **sorafenib/de** |
| **23** | **(sorafenib OR nexavar OR "bay 43 9006" OR "bay 439006" OR "bay43 9006" OR "bay43 9006" OR bay439006 OR "BAY 545 9085"):ti,ab,tn** |
| **24** | **284461-73-0:rn** |
| **25** | **bevacizumab/de** |
| **26** | **(bevacizumab OR avastin OR altuzan OR "nsc 704865" OR nsc704865):ti,ab,tn** |
| **27** | **216974 75 3:rn** |
| **28** | **axitinib/de** |
| **29** | **(axitinib OR inlyta OR "ag 013736" OR "ag 13736" OR ag013736 OR ag13736):ti,ab,tn** |
| **30** | **319460-85-0:rn** |
| **31** | **antiangiogenic therapy/de** |
| **32** | **("antiangiogenic therapy" OR "anti angiogenic therapy" OR "antiangiogenic drugs" OR "anti angiogenic drugs"):ti,ab** |
| **33** | **("vascular endothelial growth factor" OR "antivascular endothelial growth factor" OR vegf OR vegfs OR antivegf OR antivegfs OR vegfr):ti,ab** |
|  |  |
| INTERVENTION – First / Second line therapy terms | |
| **1** | **((first OR 1st OR second OR 2nd OR subsequent) NEXT/1 (line OR lines)):ti,ab** |
| **2** | **((prior OR previous OR initial) NEXT/1 (therapy OR treatment OR therapies)):ti,ab** |
| **3** | **((prior OR previous OR initial OR after OR one OR 1) NEXT/1 (other OR "vascular endothelial growth factor" OR "antivascular endothelial growth factor" OR "anti vascular endothelial growth factor" OR "anti VEGF" OR "anti VEGFs" OR "antiVEGFs" OR "antiVEGF" OR VEGF OR VEGFs OR vegfr)):ti,ab** |
| **4** | **((prior OR previous OR initial OR after OR one OR 1) NEXT/1 (lenvatinib OR cabozantinib OR nivolumab OR temsirolimus OR evorolimus OR pazopanib OR sunitinib OR sorafenib OR bevacizumab OR axitinib)):ti,ab** |
| **5** | **("after one" OR "after 1" OR "after first" OR "after initial" OR "previously treated" OR "previously received" OR pretreated OR "pre treated"):ti,ab** |
| **6** | **resistance:ti,ab** |
|  |  |
| **Cochrane and Pubmed database searches** | |
| **1** | **This strategy was modified and repeated in the Cochrane Library and PubMed databases. Slight changes to the syntax were required depending on each search platform, and to adapt indexing differences in the databases.** |
| **2** | **Cochrane Library was searched for systematic reviews, controlled trials, economic evaluations, health technology assessments and publications using the search terms for the disease drug treatments specified in the search terms. There were no limits by date and no database restrictions.** |
| **3** | **PubMed was searched for epub ahead of print and other non-MEDLINE indexed articles using search terms for the disease drug treatments specified in the search terms. There were no limits by date. Database restrictions included English language articles not indexed for MEDLINE or OLDMEDLINE and records for English language articles assigned as in process or with “pubmednotmedline” status** |
|  |  |
| **Other trial registry database searches** | |
| **1** | **Other registries searched included the U.S. NIH ClinicalTrials.gov registry, ISRCTN Registry, the UKCTG and the WHO ICTRP Search Portal. These sites were searched to identify clinical trials using the search terms for the disease drug treatments specified in the search terms. There were no limits by date and no restrictions by recruitment status of studies.** |
