# Supplementary material for: Indirect treatment comparisons including network meta-analysis: Lenvatinib plus everolimus for the second-line treatment of advanced/metastatic renal cell carcinoma
Source: PLoS One. 2019 Mar 5;14(3):e0212899. doi: 10.1371/journal.pone.0212899 (PMC6400440; doi:10.1371/journal.pone.0212899)
Supplement: S3 Table — *TARGET added after SLR, during the creation of the Bucher ITC network. (DOCX) [file pone.0212899.s005.docx]

**S3 Table. List of trials included in the ITCs.**

| **Treatment** | **Trial** | **References** |
| --- | --- | --- |
| **Everolimus controlled trials** | | |
| **Lenvatinib plus Everolimus**  **VS**  **Everolimus** | HOPE 205 | Motzer RJ, Hutson TE, Glen H, et al. (2015). “Lenvatinib, everolimus, and the combination in patients with metastatic renal cell carcinoma: A randomised, phase 2, open-label, multicentre trial.” Lancet Oncol 16, 1473-1482. |
|  | *One prior VEGF* | Motzer RJ, Hutson TE, Ren M, et al. (2016) “Independent assessment of lenvatinib plus everolimus in patients with metastatic renal cell carcinoma.” Lancet Oncol 17, e4-5. |
|  |  |  |
|  |  |  |
| **Nivolumab**  **VS**  **Everolimus** | CHECKMATE-025 | Motzer RJ, Escudier B, McDermott DF, et al. (2015). “Nivolumab versus Everolimus in Advanced Renal-Cell Carcinoma.” N Engl J Med 373, 1803-1813. |
|  |  |  |
|  | *One or two prior antiangiogenic therapies* | Motzer RJ, Sharma P, McDermott DF, et al (2016). “CheckMate 025 phase III trial: Outcomes by key baseline factors and prior therapy for nivolumab (NIVO) versus everolimus (EVE) in advanced renal cell carcinoma (RCC).” J Clin Oncol 34, (suppl 2S; abstr 498). |
|  |  |  |
| **Cabozantinib**  **VS**  **Everolimus** | METEOR | Choueiri TK, Escudier B, Powles T, et al. (2015). “Cabozantinib versus everolimus in advanced renal-cell carcinoma.” N Engl J Med 373, 1814-1823. |
|  | *At least one prior VEGF* | Choueiri TK, Escudier B, Powles T, et al. (2016). “Cabozantinib versus everolimus in advanced renal cell carcinoma (METEOR): final results from a randomised, open-label, phase 3 trial.” Lancet Oncol 17(7): 917-927. |
| **Everolimus VS**  **Placebo** | RECORD-1 | Motzer RJ, Excudier B, Oudard S, et al. (2008). “Efficacy of everolimus in advanced renal cell carcinoma: a double-blind, randomised, placebo-controlled phase III trial”. The Lancet 372: 449-456. |
|  | *Prior sorafenib and/or sunitinib* | Motzer RJ, Escudier B, Oudard S, et al. (2010). “Phase 3 trial of everolimus for metastatic renal cell carcinoma: final results and analysis of prognostic factors.” Cancer 116, 4256-4265. |
|  |  | Calvo E, Escudier B, Motzer RJ, et al. (2012). “Everolimus in metastatic renal cell carcinoma: Subgroup analysis of patients with 1 or 2 previous vascular endothelial growth factor receptor-tyrosine kinase inhibitor therapies enrolled in the phase III RECORD-1 study”. Eur J Cancer 48, 333-339. |
|  |  | Korhonen P, Zuber E, Branson M et al (2012) “Correcting overall survival for the impact of crossover via a Rank-Preserving Structural Failure Time (RPSFT) model in the RECORD-1 trial of everolimus in metastatic renal-cell carcinoma.” J Biopharm Stat 22(6): 1258-1271. |
| **Sorafenib trials** | | |
| **Axitinib**  **VS**  **Sorafenib** | AXIS | Rini BI, Escudier B, Tomczak P, et al. (2011). “Comparative effectiveness of axitinib versus sorafenib in metastatic renal cell carcinoma (AXIS): a randomised phase 3 trial.” Lancet 378, 1931-1939. |
|  | *One prior systemic treatment (cytokine or VEGF)* | Motzer RJ, Escudier B, Tomczak P, et al. (2013). “Axitinib versus sorafenib as second-line treatment for advanced renal cell carcinoma: overall survival analysis and updated results from a randomised phase 3 trial.” Lancet Oncol 14(6): 552-562. |
|  |  |  |
| **Sorafenib**  **VS**  **Placebo** | TARGET* | Escudier B, Eisen T, Stadler WM, et al (2007) “Sorafenib in advanced clear-cell renal-cell carcinoma”.N Engl J Med 2007;356:125-34. |
|  | *Prior systemic treatment (cytokines)* | Escudier B, Eisen T, Stadler WM, et al (2009) “Sorafenib for Treatment of Renal Cell Carcinoma: Final Efficacy and Safety Results of the Phase III Treatment Approaches in Renal Cancer Global Evaluation Trial” J Clin Oncol 27:4068-75. |

*TARGET added after SLR, during the creation of the Bucher ITC network
