# Supplementary material for: Indirect treatment comparisons including network meta-analysis: Lenvatinib plus everolimus for the second-line treatment of advanced/metastatic renal cell carcinoma
Source: PLoS One. 2019 Mar 5;14(3):e0212899. doi: 10.1371/journal.pone.0212899 (PMC6400440; doi:10.1371/journal.pone.0212899)
Supplement: S4 Table — (DOCX) [file pone.0212899.s006.docx]

**S4 Table. Excluded studies.**

| **Treatment** | **Trial** | **References** | **Reason for exclusion** |
| --- | --- | --- | --- |
| **Sorafenib trials** | | |  |
| **Axitinib** | Qin 2015 | Qin S, Bi F, Jin J, Cheng Y, et al. (2015) “Axitinib versus sorafenib as a second-line therapy in Asian patients with metastatic renal cell carcinoma: results from a randomised registrational study.” Onco Targets Ther 8: 1363-1373. | Less comparable patient population than pivotal AXIS trial (Asian, less prior VEGF). |
| **Placebo** | Ratain 2006 | Ratain MJ, Eisen T, Stadler WM, et al. (2006) “Phase II Placebo-Controlled Randomized Discontinuation Trial of Sorafenib in Patients With Metastatic Renal Cell Carcinoma” J Clin Oncol 24(16):2505-2512. | Randomised discontinuation design. Limited reporting of outcomes (No OS). |
| **Temsirolimus** | INTORSECT | Hutson et al. “Randomized Phase III Trial of Temsirolimus Versus Sorafenib As Second-Line Therapy After Sunitinib in Patients With Metastatic Renal Cell Carcinoma” Journal of Clinical Oncology 2014 32:8, 760-767 | Not a relevant comparison.  Patients had failed sunitinib only, associated with poorer outcomes. |
| **Sunitinib** | SWITCH | Eichelberg, Christian, et al. "SWITCH: a randomised, sequential, open-label study to evaluate the efficacy and safety of sorafenib-sunitinib versus sunitinib-sorafenib in the treatment of metastatic renal cell cancer." European urology 68.5 (2015): 837-847. | Not a relevant comparison.  Cross-over trial comparing sequential treatment of SOR followed by SUN and vice-versa |
