# Supplementary material for: Indirect treatment comparisons including network meta-analysis: Lenvatinib plus everolimus for the second-line treatment of advanced/metastatic renal cell carcinoma
Source: PLoS One. 2019 Mar 5;14(3):e0212899. doi: 10.1371/journal.pone.0212899 (PMC6400440; doi:10.1371/journal.pone.0212899)
Supplement: S5 Table — (DOCX) [file pone.0212899.s007.docx]

**S5 Table. Randomized controlled trials quality assessment**

| **Study** | **Selection Bias** | | | **Performance Bias** | | | **Attrition Bias** | | | **Detection Bias** | | | | |
| --- | --- | --- | --- | --- | --- | --- | --- | --- | --- | --- | --- | --- | --- | --- |
|  | Appropriate method of randomization | Adequate concealment of allocation? | Groups comparable at baseline? | Comparison groups received the same care? | Participants receiving care were kept blind? | Individuals receiving care were kept blind? | All groups followed for an equal amount of time? | Groups were comparable for treatment completion? | Groups were comparable with respect to the availability of out | Study had an appropriate length of follow-up? | Study had a precise definition of outcome? | A valid and reliable method was used to determine the outcome? | Investigators were kept blind to the participants exposure | Investigators were kept blind to other confounding and prognostic factors? |
| **HOPE** | Yes | N/A- open label | Yes- except patients with ≥3 metastases, patients who received SUN | Yes | No | No but outcomes independently assessed | Yes | Yes | Yes | Yes | Yes | Yes | Not clear | Not clear |
| **CHECKMATE-025** | Yes | N/A- open label | Yes | Not clear | Yes | Yes | No | No | Yes | Yes | Yes | Yes | Yes | Yes |
| **METEOR** | Yes | N/A- open label | Yes | N/A- open label | Yes | Yes | No | No but outcomes independently assessed | Yes | Yes | Yes | Yes | Yes | Yes |
| **RECORD-1** | Yes | Yes | Yes | Yes | Yes | Yes | Yes | Yes | Yes | Yes | Yes | Not clear | Yes | Yes |
| **AXIS** | Yes | N/A- open label | Yes | Yes | Yes | Yes, AXI patients without hypertension and good tolerability could increase their doses and SUN could not | No | No but outcomes independently assessed | Yes | Yes | Yes | Yes | Yes | Yes |
