# Supplementary material for: Indirect treatment comparisons including network meta-analysis: Lenvatinib plus everolimus for the second-line treatment of advanced/metastatic renal cell carcinoma
Source: PLoS One. 2019 Mar 5;14(3):e0212899. doi: 10.1371/journal.pone.0212899 (PMC6400440; doi:10.1371/journal.pone.0212899)
Supplement: S6 Table — *The category Unknown signified unreported, missing, or unknown baseline characteristic data as reported by the original trial. ECOG, Eastern Cooperative Oncology group; MSKCC, Memorial Sloan-Kettering Cancer Center; NR, not reported; RT, radiotherapy; VEGF, vascular endothelial growth factor; HOPE 205, METEOR, TARGET and AXIS reported ECOG performance status; CHECKMATE-025 and RECORD-1 reported Karnofsky performance. (DOCX) [file pone.0212899.s008.docx]

S6 Table: Baseline patient and disease characteristics. *The category *Unknown* signified unreported, missing, or unknown baseline characteristic data as reported by the original trial.

|  | **HOPE 205** | | **CHECKMATE-025** | | | **METEOR** | | **RECORD-1** | | **TARGET** | | | **AXIS** | |
| --- | --- | --- | --- | --- | --- | --- | --- | --- | --- | --- | --- | --- | --- | --- |
|  | **LEN + EVE (N=51)** | **Evero-limus (N=50)** | **Nivo-lumab (N=410)** | **Evero-limus (N=411)** | | **Caboz-antinib (N=330)** | **Evero-limus (N=328)** | **Place-bo (N=139)** | **Evero-limus (N=277)** | **Place-bo (N=452)** | **Sora-fenib (N=451)** | | **Axi-tinib (N=361)** | **Sora-fenib (N=362)** |
| **Age, median (range)** | 61 (44 - 79) | 59 (37 – 77) | 62 (23 – 88) | 62 (18 – 86) | | 63 (32 – 86) | 62 (31 – 84) | 60 (29 – 79) | 61 (27 – 85) | 59 (29 – 84) | 58 (19 – 86) | | 61 (20 – 82) | 61 (22 – 80) |
| **Gender** |  |  |  |  | |  |  |  |  |  |  | |  |  |
| **Male** | 69% | 76% | 77% | 74% | | 77% | 73% | 76% | 78% | 75% | 70% | | 71% | 73% |
| **Race** |  |  |  |  | |  |  |  |  |  |  | |  |  |
| **White** | NR | NR | 86% | 89% | | 82% | 80% | NR | NR | NR | NR | | 77% | 74% |
| **Asian** | NR | NR | 10% | 8% | | 6% | 8% | NR | NR | NR | NR | | 21% | 22% |
| **Black** | NR | NR | <1% | 1% | | 2% | <1% | NR | NR | NR | NR | | <1% | 1% |
| **Unknown*** | NR | NR | 3% | 2% | | 11% | 11% | NR | NR | NR | NR | | 1% | 3% |
| **ECOG/Karnofsky performance status^a^** | | | | | | | | | |  | | | | |
| **0/90-100** | 53% | 56% | 68% | | 65% | 68% | 66% | 68% | 63% | 46% | 49% | | 54% | 55% |
| **1/70-80** | 47% | 44% | 32% | | 35% | 32% | 34% | 33% | 36% | 52% | 49% | | 45% | 44% |
| **2/<70** | - | - | <1% | | <1% | - | - | - | - | 1% | 2% | | <1% | 0% |
| **MSKCC risk** |  |  |  | |  |  |  |  |  |  |  | |  |  |
| **Favourable** | 24% | 24% | 35% | | 36% | 45% | 46% | 28% | 29% | 50% | 52% | | 28% | 28% |
| **Intermediate** | 37% | 38% | 49% | | 49% | 42% | 41% | 57% | 56% | 49% | 48% | | 37% | 36% |
| **Poor** | 39% | 38% | 16% | | 15% | 12% | 13% | 15% | 14% | - | - | | 33% | 33% |
| **Unknown*** | - | - | - | | - | - | - | - | - | <1% | - | | 2% | 3% |
| **Prior VEGF** |  |  |  | |  |  |  |  |  |  | | | | |
| **1** | 100% | 100% | 72% | | 72% | 71% | 70% | 74% | 74% | 0% | 0% | | 65% | 65% |
| **≥2** | 0% | 0% | 28% | | 28% | 29% | 30% | 26% | 26% | 0% | 0% | | 0% | 0% |
| **SUN** | 71% | 56% | 60% | | 59% | 64% | 62% | 69% | 71% | 0% | 0% | | 54% | 54% |
| **Prior Cytokine** | 0% | 0% | 0% | | 0% | 0% | 0% | 0% | 0% | 81% | 83% | | 35% | 35% |
| **Prior RT** | 12% | 22% | NR | | NR | 33% | 33% | 27% | 31% | 24% | 27% | | NR | NR |
| **Prior nephrectomy** | 86% | 96% | 89% | | 87% | 85% | 85% | 96% | 97% | 93% | | 94% | 91% | |

ECOG, Eastern Cooperative Oncology group; MSKCC, Memorial Sloan-Kettering Cancer Center; NR, not reported; RT, radiotherapy; VEGF, vascular endothelial growth factor; HOPE 205, METEOR, TARGET and AXIS reported ECOG performance status; CHECKMATE-025 and RECORD-1 reported Karnofsky performance.
