# Supplementary material for: Indirect treatment comparisons including network meta-analysis: Lenvatinib plus everolimus for the second-line treatment of advanced/metastatic renal cell carcinoma
Source: PLoS One. 2019 Mar 5;14(3):e0212899. doi: 10.1371/journal.pone.0212899 (PMC6400440; doi:10.1371/journal.pone.0212899)
Supplement: S7 Table — NR, not reported; VEGF, vascular endothelial growth factor; LEN, lenvatinib; EVE, everolimus. (DOCX) [file pone.0212899.s009.docx]

S7 Table: Subsequent therapies.

| Trial | Control patients crossover to investigational treatment | Continued treatment with study drug after progression | Subsequent systemic therapies | |
| --- | --- | --- | --- | --- |
| Everolimus trials |  |  | **Treatment** | **Everolimus** |
| HOPE 205 (LEN + EVE) | Not permitted | Not permitted | Any: 28%  Any VEGF: 16%  Axitinib 12%  everolimus 10% | Any: 36%  Any VEGF 26%  Axitinib 24%  everolimus 4% |
| CHECKMATE-025  (nivolumab) | Not permitted | NR | Any: 55%  everolimus 26%  Axitinib 24%  Pazopanib 9% | Any: 63%  Axitinib 36%  Pazopanib 16%  Sorafenib 9% |
| METEOR (cabozantinib) | Not permitted | Treatment continued while clinical benefit was observed | Any: 50%  Any VEGF 24%  everolimus 29%  Axitinib 17% | Any: 55%  Any VEGF 47%  Axitinib 27%  SUN 10% |
| RECORD-1 (placebo) | 80% | Not permitted | NR | |
| Sorafenib trials |  |  | **Treatment** | **Sorafenib** |
| AXIS (axitinib) | Not permitted | Not permitted | Any: 54%  Any VEGF 33%  Any mTOR 39%  everolimus 16% | Any: 57%  Any VEGF 32%  Any mTOR 41%  everolimus 8% |
| TARGET (placebo) | 48% | Patients who responded could continue sorafenib | NR | |

NR, not reported; VEGF, vascular endothelial growth factor; LEN, lenvatinib; EVE, everolimus.
