# Supplementary material for: Indirect treatment comparisons including network meta-analysis: Lenvatinib plus everolimus for the second-line treatment of advanced/metastatic renal cell carcinoma
Source: PLoS One. 2019 Mar 5;14(3):e0212899. doi: 10.1371/journal.pone.0212899 (PMC6400440; doi:10.1371/journal.pone.0212899)
Supplement: S8 Table — CI, confidence interval; HR, hazard ratio; NR, not reported; PFS, progression free survival; VEGF, vascular endothelial growth factor; vs, versus. a Primary PFS population with 375 patients; b Protocol defined analysis of 769 patients; c At time of cross-over in 903 patients. (DOCX) [file pone.0212899.s010.docx]

S8 Table: Progression-free survival as reported in the individual trials.

| **Treatment** | **Prior experience** | **Data cut** | **Assessment method** | **Median PFS (months)** | | **HR (95% CI)** |
| --- | --- | --- | --- | --- | --- | --- |
| **Everolimus trials** | | | | **Treatment** | **Everolimus** | **vs Everolimus** |
| Lenvatinib plus everolimus | 1 prior VEGF | 13 Jun 2014 | Investigator | 14.6 | 5.5 | 0.40 (0.24-0.68) |
|  |  | 13 Jun 2014 | Independent | 12.8 | 5.6 | 0.45 (0.27-0.79) |
|  | Prior sunitinib | 13 Jun 2014 | Investigator | NR | NR | 0.36 (0.19-0.67) |
| Nivolumab | 1-2 prior VEGF | June 2015 | Investigator | 4.6 | 4.4 | 0.88 (0.75-1.03) |
| Cabozantinib | ≥1 prior VEGFa | 22 May 2015 | Independent | 7.4 | 3.8 | 0.58 (0.45-0.75) |
|  | Prior sunitinib | 22 May 2015 | Independent | 9.1 | 3.7 | 0.41 (NR) |
| Placebo | Prior sunitinib and/or sorafenib | 15 Oct 2007 | Independent | 1.9 | 4.0 | 3.33 (2.50-4.55) |
|  |  | 15 Oct 2007 | Investigator | 1.8 | 4.6 | 3.23 (2.44-4.17) |
|  | 1 prior VEGF | 28 Feb 2008 | Independent | 1.9 | 5.4 | 3.13 (2.33-4.17) |
|  | Prior sunitinib | 28 Feb 2008 | Independent | 1.8 | 3.9 | 2.94 (1.96-4.35) |
| **Sorafenib trials** | | | | **Treatment** | **Sorafenib** | **vs Sorafenib** |
| Axitinib | 1 prior VEGF or cytokine | 31 Aug 2010 | Independent | 6.7 | 4.7 | 0.67 (0.54-0.81) |
|  |  | 31 Aug 2010 | Investigator | 8.3 | 5.6 | 0.66 (0.54-0.80) |
|  | Prior sunitinib | 31 Aug 2010 | Independent | 4.8 | 3.4 | 0.74 (0.57, 0.96) |
|  |  | 1 Nov 2011 | Investigator | 6.5 | 4.4 | 0.72 (0.57-0.90) |
|  | Prior cytokine | 31 Aug 2010 | Independent | 12.1 | 6.5 | 0.46 (0.32-0.68) |
|  |  | 1 Nov 2011 | Investigator | 12.2 | 8.2 | 0.51 (0.37-0.68) |
| Placebo | No prior VEGFb | Jan 2005 | Independent | 2.8 | 5.5 | 2.27 (1.82-2.86) |
|  | No prior VEGFc | May 2005 | Investigator | NR | NR | 1.96 (1.67-2.33) |

CI, confidence interval; HR, hazard ratio; NR, not reported; PFS, progression free survival; VEGF, vascular endothelial growth factor; vs, versus. a Primary PFS population with 375 patients; b Protocol defined analysis of 769 patients; c At time of cross-over in 903 patients.
