# Supplementary material for: Indirect treatment comparisons including network meta-analysis: Lenvatinib plus everolimus for the second-line treatment of advanced/metastatic renal cell carcinoma
Source: PLoS One. 2019 Mar 5;14(3):e0212899. doi: 10.1371/journal.pone.0212899 (PMC6400440; doi:10.1371/journal.pone.0212899)
Supplement: S9 Table — CI, confidence interval; HR, hazard ratio; ITC, indirect treatment comparison; NR, not reported; OS, overall survival; RPSFT, rank preserving structural failure time; VEGF, vascular endothelial growth factor; vs, versus. a 98.5% confidence interval. (DOCX) [file pone.0212899.s011.docx]

S9 Table: Overall survival as reported in the individual trials.

| **Treatment** | **Prior experience** | **Data cut** | **Median follow-up for OS (months)** | **Median OS (months)** | | | | **HR (95% CI)** |
| --- | --- | --- | --- | --- | --- | --- | --- | --- |
| **Everolimus trials** | |  |  | **Treatment** | | **Everolimus** | | **vs Everolimus** |
| Lenvatinib plus everolimus | 1 prior VEGF | 31 Jul 2015 | NR | 25.5 |  |  | 19.1 | 0.59 (0.36-0.97) |
| Nivolumab | 1-2 prior VEGF | June 2015 | [minimum 14] | 25 |  |  | 19.6 | 0.73 (0.57-0.93)a |
|  | 1 prior VEGF | June 2015 | NR | NR |  |  | NR | 0.71 (0.56-0.90) |
| Cabozantinib | ≥1 prior VEGF | 31 Dec 2015 | ~18.7 | 21.4 |  |  | 16.5 | 0.66 (0.53-0.83) |
| Placebo | Prior sunitinib and/or sorafenib | 15 Nov 2008 | NR | 14.4 |  |  | 14.8 | 1.15 (0.87-1.54) |
|  |  | 15 Nov 2008 | NR |  | RPSFT | |  | 1.67 (0.61-4.55) |
| **Sorafenib trials** | |  |  | **Treatment** | | **Sorafenib** | | **vs Sorafenib** |
| Axitinib | 1 prior VEGF or cytokine | Nov 2011 | NR | 20.1 |  |  | 19.2 | 0.97 (0.80-1.18) |
|  | Prior sunitinib | Nov 2011 | NR | 15.2 |  |  | 16.5 | 1.00 (0.78-1.27) |
| Placebo | No prior VEGF | Sep 2006 | NR | 15.2 |  |  | 17.8 | 1.14 (0.96-1.35) |
|  |  | Sep 2006 | Alive placebo patients were censored at cross-over | 14.3 |  |  | 17.8 | 1.28 (1.03-1.61) |

CI, confidence interval; HR, hazard ratio; ITC, indirect treatment comparison; NR, not reported; OS, overall survival; RPSFT, rank preserving structural failure time; VEGF, vascular endothelial growth factor; vs, versus. a 98.5% confidence interval
