# Supplementary material for: Indirect treatment comparisons including network meta-analysis: Lenvatinib plus everolimus for the second-line treatment of advanced/metastatic renal cell carcinoma
Source: PLoS One. 2019 Mar 5;14(3):e0212899. doi: 10.1371/journal.pone.0212899 (PMC6400440; doi:10.1371/journal.pone.0212899)
Supplement: S10 Table — CI, confidence interval; n/N, number with event/number in efficacy population; NR, not reported; ORR, overall response rate; VEGF, vascular endothelial growth factor. (DOCX) [file pone.0212899.s012.docx]

S10 Table: Overall response rate as reported in the individual trials.

| **Treatment** | **Prior experience** | **Data cut** | **Assessment method** |  |  | | **ORR, n/N (%)** | |  |
| --- | --- | --- | --- | --- | --- | --- | --- | --- | --- |
| **Everolimus trials** | |  |  |  | | **Treatment** | | **Everolimus** | |
| Lenvatinib plus | 1 prior VEGF  1 prior VEGF | 13 Jun 2014  13 Jun 2014 | Investigator  Independent |  | | 22/51 (43%)  18/51 (35%) | | 3/50 (6%)  0/50 (0%) | |
|  |  |  |  |  | |  | |  | |
|  | Prior sunitinib | 13 Jun 2014 | Investigator | NR |  | |  |  | NR |
| Nivolumab | 1-2 prior VEGF | June 2015 | Investigator |  | | 103/410 (25%) | | 22/411 (5%) | |
|  | 1 prior VEGF | June 2015 | Investigator |  | | NR (24%) | | NR (5%) | |
| Cabozantinib | ≥1 prior VEGF | 22 May 2015 | Independent |  | | 57/330 (17%) | | 11/328 (3%) | |
|  | Prior sunitinib | 22 May 2015 | Independent |  | | 17/76 (22%) | | 2/77 (3%) | |
| Placebo | Prior sunitinib and/or sorafenib | 28 Feb 2008 | Independent |  | | 0/138 (0%) | | 5/272 (2%) | |
| **Sorafenib trials** | |  |  |  | | **Treatment** | | **Sorafenib** | |
| Axitinib | 1 prior VEGF or cytokine | 31 Aug 2010 | Independent |  | | 70/361 (19%) | | 34/362 (9%) | |
|  | 1 prior VEGF or cytokine | 1 Nov 2011 | Investigator |  | | 82/361 (23%) | | 45/362 (12%) | |
| Placebo | No prior VEGF | Jan 2005 | Independent |  | | 0/337 (0%) | | 7/335 (2%) | |
|  | No prior VEGF | May 2005 | Investigator |  | | 8/452 (2%) | | 44/451 (10%) | |

CI, confidence interval; n/N, number with event/number in efficacy population; NR, not reported; ORR, overall response rate; VEGF, vascular endothelial growth factor.
