# Supplementary material for: Indirect treatment comparisons including network meta-analysis: Lenvatinib plus everolimus for the second-line treatment of advanced/metastatic renal cell carcinoma
Source: PLoS One. 2019 Mar 5;14(3):e0212899. doi: 10.1371/journal.pone.0212899 (PMC6400440; doi:10.1371/journal.pone.0212899)
Supplement: S11 Table — *Indicates significance at a 5% significance level CI confidence interval; LEN, lenvatinib; EVE, everolimus; EMA, European Medicines Agency; FDA, Food and Drug Administration. (DOCX) [file pone.0212899.s013.docx]

**S11** **Table. Overall Survival applying ITT results from TARGET and RECORD-1**

| **OS - ITT** | | | |
| --- | --- | --- | --- |
| **LEN + EVE vs** | **EMA (2016)** | **FDA (2016)** | **Motzer (2015)** |
| **Placebo** | 0.51 (0.29 ; 0.90)* | 0.58 (0.33 ; 1.01) | 0.44 (0.24 ; 0.81)* |

*Indicates significance at a 5% significance level CI confidence interval; LEN, lenvatinib; EVE, everolimus; EMA, European Medicines Agency; FDA, Food and Drug Administration.
