# Supplementary material for: Indirect treatment comparisons including network meta-analysis: Lenvatinib plus everolimus for the second-line treatment of advanced/metastatic renal cell carcinoma
Source: PLoS One. 2019 Mar 5;14(3):e0212899. doi: 10.1371/journal.pone.0212899 (PMC6400440; doi:10.1371/journal.pone.0212899)
Supplement: S2 File — (PDF) [file pone.0212899.s015.pdf]

PCN24

Comparing ITC Results From Lenvatinib Plus Everolimus for Second-line Treatment of Advanced/Metastatic Renal Cell Carcinoma: Crossover Versus No Crossover

Gabriel Tremblay[1], Shan Ashton Garib[1], Genevieve Meier[2], Heather McElroy[3], Matthew Guo[2], Anna Forsythe[1]

[1]Purple Squirrel Economics, New York, NY; [2]Eisai Inc., Woodcliff Lake, NJ; [3]Covance (Asia) Pte Ltd, Singapore, Singapore

BACKGROUND

- Globally, about 270,000 cases of kidney cancer are diagnosed yearly and 116,000 people die from the disease.[1]
- HOPE 205 a phase II, multi-center study comparing LEN + EVE against EVE alone showed that the combination of LEN and EVE prolonged the PFS (14.6 months vs. 5.5 months; HR: 0.40; 95% CI: 0.24-0.68; p<0.001) and OS (25.5 months vs. 15.4 months; HR: 0.51; 95% CI: 0.30-0.88; p=0.024) as compared to EVE alone in RCC patients following progression after 1 prior anti–vascular endothelial growth factor (VEGF) targeted therapy.[2]
- The combination of LEN and EVE was approved by U. S. Food and Drug Administration in 2016, in combination with everolimus, for the treatment of advanced renal cell carcinoma following one prior anti-angiogenic therapy. It was also accepted for priority review by the European Medicines Agency (EMA) for the second line treatment of mRCC.
- However, in the absence of randomized controlled trials involving a direct (head-to-head) comparison of all second line treatments for RCC, an indirect treatment comparison (ITC) involving lenvatinib plus everolimus (LEN+EVE) was conducted using networked data from HOPE 205[2], CHECKMATE-025[5] (Motzer 2015, nivolumab vs everolimus), METEOR[4] (Choueiri 2015, cabozantinib vs everolimus), AXIS[3] (Motzer 2013, AXI vs SOR), RECORD-1[7] (Motzer 2008, everolimus vs PBO) and TARGET[6] (Escudier 2009, sorafenib vs PBO), Motzer 2013[8] (pazopanib vs sunitinib) and Hainsworth 2013[9] (pazopanib vs PBO).
- The ITC incorporated adjustments for crossover to investigational treatment.
- In trials where patients switch from a less effective to more effective treatment arms, a bias is observed in underestimated results not adjusted for treatment crossover.

OBJECTIVES & METHODS

- A follow-up analysis using updated HOPE 205 data (July 2015) with intention to treat (ITT) data versus adjusted-ITT data evaluated the impact of crossover correlation on OS estimates and additionally explored potential bias due to the absence of adjustment. RECORD-1 (EVE vs PBO) and TARGET (SOR vs PBO) allowed 80% and 48% patient crossover, respectively. Hainsworth (PAZ vs PBO) also allowed patient crossover (RPSFT adjusted)
- Three ITC scenarios were analyzed using a classic frequentist ITC using the Bucher (1997) method with a two-sided 95% confidence interval and the epidemiological endpoints were evaluated in terms of hazard ratios for OS and PFS:
  - Scenario "A": all comparators plus placebo versus EVE.
  - Scenario "B": all comparators versus placebo.
  - Scenario "C": LEN+EVE versus all comparators + placebo.

RESULTS

Scenario A - EMA (2016) data cut

- Hazard ratios for OS in AXI vs. EVE shifted from below null (0.98) to above null (1.27); and mortality risk (placebo vs. EVE) moved 51% further from null (1.15 vs. 1.67); A shift further away from the null suggests higher motility risk.
- Scenario "A" showed a wide range in OS HR change after adjusting for crossover: -31.4% (PAZ vs EVE) to +45.2% (PBO vs EVE)
- Further, in "A," three of the HR values increased (SOR, AXI, PBO) and two decreased (PAZ, SUN)

Scenarios B - EMA (2016) data cut

- Scenario "B" shows all ITT HR values for OS closer to null than with the crossover adjusted population
- Also, the direction of HR change is negative for all treatments and ranges from: -10% (AXI vs PBO) to -53.0% (SUN vs PBO)

Scenario C - EMA (2016) data cut

- Scenario "C" showed a wide range in change for HR values after crossover adjustment: -22.4% (LEN+EVE vs SOR) to +47.1% (LEN+EVE vs SUN)
- Further, in "C" two of the values increased (PAZ, S"UN) and three decreased (SOR, AXI, PBO).

CONCLUSIONS

- Bias was observed in naive approaches to survival analysis in the presence of crossover. Failure to account for this in clinical trials may have implications on the comparative effectiveness profile and also on the cost-effectiveness results and may lead to inconsistent resource allocation decisions.

Acknowledgement:
Study sponsored by Eisai
Contact: AnnaForsythe@pshta.com
+1-646-478-8213

Table 1: Scenario A

Treatment (*Significant at 5%)	Comp-arator	OS		
		ITT Population	Crossover Adjusted Population	% Change
LEN+EVE	EVE	0.59 (0.36-0.97)	NA	NA
PAZ		1.05 (0.72-1.53)	0.72 (0.18-2.89)	-31.4%
SOR		1.01 (0.72-1.41)	1.30 (0.46-3.64)	28.7%
SUN		1.15 (0.76-1.74)	0.79 (0.19-3.20)	-31.3%
AXI		0.98 (0.67-1.44)	1.27 (0.45-3.62)	29.6%
CAB		0.66 (0.53-0.83)*	NA	NA
NIV		0.73 (0.57-0.93)*	NA	NA
PBO		1.15 (0.87-1.54)	1.67 (0.61-4.55)	45.2%

Table 2: Scenario B

Treatment (*Significant at 5%)	Comp-arator	OS (ITT population)	OS (Crossover Adjusted Population)	% Change
LEN+EVE	PBO	0.51 (0.29-0.90)*	0.35 (0.11-1.07)	-31.4%
PAZ		0.91 (0.57-1.46)	0.43 (0.08-2.39)	-52.7%
SOR		0.88 (0.57-1.37)	0.78 (0.18-3.30)	-11.4%
SUN		1.00 (0.60-1.65)	0.47 (0.08-2.66)	-53.0%
AXI		0.85 (0.53-1.37)	0.76 (0.18-3.23)	-10.6%
CAB		0.57 (0.40-0.82)*	0.40 (0.14-1.12)	-29.8%
NIV		0.63 (0.43-0.92)*	0.44 (0.16-1.24)	-30.2%

Table 3: Scenario C

Treatment (*Significant at 5%)	Comp-arator	OS		
		ITT Population	Crossover Adjusted Population	% Change
LEN+EVE	EVE	0.59 (0.36-0.97)*	0.59 (0.36-0.97)*	0%
	PAZ	0.56 (0.30-1.04)	0.82 (0.19-3.58)	46.4%
	SOR	0.58 (0.32-1.06)	0.45 (0.14-1.42)	-22.4%
	SUN	0.51 (0.27-0.97)*	0.75 (0.17-3.35)	47.1%
	AXI	0.60 (0.32-1.12)	0.46 (0.15-1.46)	-23.3%
	CAB	0.89 (0.52-1.53)	0.89 (0.52-1.53)	0%
	NIV	0.81 (0.47-1.41)	0.81 (0.47-1.41)	0%
	PBO	0.51 (0.29-0.90)*	0.35 (0.11-1.07)	-31.4%

LIMITATIONS

- The current method assumes that for patients A and B, if patient A has an event before patient B on a particular treatment then patient A would also have the event before patient B if both were to follow an alternative treatment
- The method also assumes that the treatment effect is the same for patients who switched in the control arm as for the experimental arm
- The model is based explicitly on the groups as randomized and estimates a treatment effect, which is only statistically significant if the ITT analysis is statistically significant
- In practice, it is advisable to assess the robustness of the crossover adjustment by applying other methods commonly used

REFERENCES

1. Global Data: EpiCast, "Renal Cell Carcinoma - Epidemiology Forecast To 2023," [Online]. Available: http://www.marketresearch.com/product/sample-8353546.pdf. [Accessed 18 Jan 2016].
2. Motzer et. al, "Randomized phase II, three-arm trial of lenvatinib (LEN), everolimus (EVE), and LEN+ EVE in patients (pts) with metastatic renal cell carcinoma (mRCC).," in ACSO Annual Meeting, 2015
3. Motzer et. al, Axitinib vs. Sorafenib as second line treatment for advanced renal cell carcinoma: overall survival analysis and updated results from a randomized phase 3 trial Lancet Oncol. 2013 May;14(6):552-62
4. Choueiri et. al, Cabozantinib versus Everolimus in Advanced Renal-Cell Carcinoma N Engl J Med Nov 2015; 373:1814-1823
5. Motzer et. al, Nivolumab versus Everolimus in Advanced Renal-Cell Carcinoma N Engl J Med Nov 2015; 373:1803-1813
6. Motzer et. al, Pazopanib versus Sunitinib in Metastatic Renal-Cell Carcinoma N Engl J Med. 2013 Aug 22;369(8):722-31
7. Escudier et. al, Sorafenib for Treatment of Renal Cell Carcinoma: Final Efficacy and Safety Results of the Phase III Treatment Approaches in Renal Cancer Global Evaluation Trial J Clin Oncol. 2009 Jul 10;27(20):3312-8.
8. Motzer et al, Pazopanib versus Sunitinib in Metastatic Renal Cell-Cell Carcinoma. N Engl J Med. 2013 Aug 22;369(8):722-31
9. Hainsworth JD, Rubin MS, Arrowsmith ER, Khatcheressian J, Crane EJ, Franco LA. Pazopanib as second-line treatment after sunitinib or bevacizumab in patients with advanced renal cell carcinoma a Sarah Cannon oncology research consortium phase II trial. Clin Genitourin Cancer. 2013;11:270-275
